# Chain Reaction of Fenton Autoxidation of Tartaric Acid: Critical Behavior at Low pH

**DOI:** 10.1021/acs.jpcb.3c02172

**Published:** 2023-05-10

**Authors:** Robert E. Coleman, Roger B. Boulton, Alexei A. Stuchebrukhov

**Affiliations:** † Department of Viticulture and Enology, University of California, Davis, California 95616, United States; ‡ Department of Chemistry, University of California, Davis, California 95616, United States

## Abstract

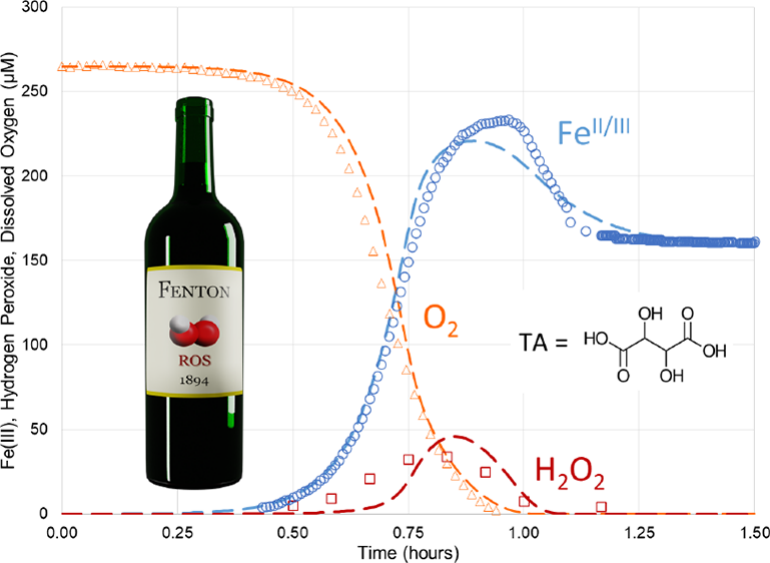

Autoxidation of tartaric acid in air-saturated aqueous solutions in the presence of Fe(II) at low pH, 2.5, shows autocatalytic behavior with distinct initiation, propagation, and termination phases. With increasing pH, the initiation phase speeds up, while the propagation phase shortens and reduces to none. We show that the propagation phase is a chain reaction that occurs via activation of oxygen in the initiation stage with the production of hydrogen peroxide. The subsequent Fenton oxidation that regenerates hydrogen peroxide with a positive feedback is typical of a self-sustained chain reaction. The conditions for such a chain reaction are shown to be similar to those of a dynamical system with critical behavior; namely, the system becomes unstable when the kinetic matrix of pseudo-first-order reaction becomes negatively defined with a negative eigenvalue giving the rate of exponential (chain) growth of the reactive species.

## Introduction

The oxidation of tartaric acid by the addition of Fe(II) and hydrogen peroxide (H_2_O_2_) was first reported by Fenton in 1876 and described in detail two decades later.^1,2^ This simple reaction played a prominent role in the development of modern chemistry and remarkably continues to be relevant today,^3−5^ in particular for wine oxidation^6^ and in general for food chemistry.

“Fenton reaction” is associated with the key reaction between Fe(II) ions and H_2_O_2_, which generates reactive oxygen species, hydroxyl radical (OH·), and/or ferryl ion (FeO^2+^), which produce secondary oxidation of various organic compounds (Fenton chemistry). The exact mechanism is still debated,^7−15^ as the prevailing intermediate depends on the conditions of the reaction: pH,^16,17^ chelates,^18−20^ and other components of the reaction. The importance of the reactive oxygen species generated by Fenton reaction in various branches of chemistry and biology is well documented.^21−24^

What is interesting is that oxidation of tartaric acid in the presence of Fe(II) can also occur without the addition of hydrogen peroxide. Here, “activation” of dissolved oxygen by iron generates hydrogen peroxide in the solution internally, which eventually is responsible for Fenton “autoxidation.”^25,26^ Tartaric acid, in this regard, is unique as it both can act as a catalyst for oxygen activation and has unique oxidation properties in a group of similar acids: malic, succinic, citric, etc.^27,28^

Recently,^27,28^ we have described the kinetics of tartaric acid autoxidation at low pH, 2.5 to 4.5, relevant to wine conditions and shown that it is quite remarkable, demonstrating both activation and chain-like propagation stages separately. The involvement of tartrate-iron complexes of yet unknown nature plays a key role in the catalysis of the reaction. The reaction kinetics were studied by measuring dissolved oxygen consumption and monitoring the status of Fe(II)/Fe(III) and H_2_O_2_ pool in the solution. The fast, chain-like oxidation kinetic phase is pH-dependent and not present at higher pH.

Here, we show that the presence or absence of the chain oxidation phase in oxidation kinetics is related to stability of the kinetic matrix of the system and is similar to the critical behavior of a dynamical system with a phase transition; namely, the chain oxidation occurs when the kinetic matrix of pseudo-first-order kinetic equations describing reactive radicals and hydrogen peroxide acquires one negative eigenvalue. At low pH, this gives rise to exponential growth (inflation) of a radical concentration, which in turn is stabilized by a nonlinear radical dimerization reaction; together, the two competing processes result in a stable propagation reaction observed in the kinetics of Fenton autoxidation. The radically different kinetic behavior of the oxidation system at high pH does not show such instability and is characterized by the absence of the exponential growth phase. Thus, under certain conditions, Fenton oxidation occurs as a controlled chain reaction.

## Materials and Methods

The experimental data were obtained as described previously.^28,29^ Briefly, the oxidation reaction of air-saturated tartaric acid (TA = COOH-(HCOH)_2_-COOH) mixed with concentrated Fe(II) sulfate was studied. An oxygen analyzer was used to measure dissolved oxygen during kinetic reactions. Direct spectrophotometric measurements of Fe(III) were taken to probe the Fe(II)/Fe(III) content. A dye and pH modification to the decolorization assay allowed for quantification of hydrogen peroxide in tartaric acid/Fe(II) solutions. To understand the kinetics observed and the underlying chemical mechanism, catalase, SOD, hydrogen peroxide, and Fe(III) chloride were systematically added to the base conditions. The kinetic modeling and resulting theoretical fits were produced with Kintecus software.^30^

The purpose of the present work is to extend theoretical analysis of the obtained experimental results and obtain deeper insights into the underlying mechanisms of the reaction. This is achieved by reducing theoretical description to a few principal components of the reaction: tartaric acid radicals, hydrogen peroxide, Fe(II)/Fe(III), and oxygen concentrations. We introduced pseudo-first-order reaction constants and analyzed the stability of the resulting pseudo-first-order reaction kinetic scheme as a function of time and pH. This approach reveals the presence or absence, at different pH, of the exponential growth of the initially small concentrations of radicals, characteristic of a chain multiplication reaction. The exponential growth of radicals (inflation) is indicated by the negative eigenvalue of the pseudo-first-order kinetic matrix, for which an analytical solution is obtained. The presence or absence of the negative kinetic eigenvalues at different pH resembles the critical behavior of dynamical systems with phase transitions; this analogy provides additional insights into the mechanism of autoxidation of air-saturated Fe(II)/tartaric acid solutions.

## Results and Discussion

### Autocatalytic Nature of Oxidation

The consumption of dissolved oxygen in an air-saturated solution of Fe(II) and tartaric acid displays a very distinct autocatalytic character with clearly defined initiation, propagation, and termination phases^27,28^ (see figures below and in the Supporting Information). At low pH, the autocatalytic curve displays approximately linear propagation with time, indicating zero-order kinetics to both oxygen and Fe(II). This feature disappears at high pH, 4.5. Changing the pH produced prominent changes in the three phases of the autocatalytic curve from pH 2.5 to 4.5. The lag phase decreased with increasing pH across the pH levels. Although pH 2.5 and 3.0 were similar in propagation and extent of oxygen consumed, from pH 3.0 to 4.5, the propagation and extent of reaction decreased with increasing pH level. A detailed report on these experiments can be found elsewhere.^27,28^ This paper will address the underlying mechanisms. The qualitative picture of what is observed is as follows.

At low pH, two phases of oxidation kinetics, initiation and propagation, are clearly seen. According to our model, the first phase is oxygen activation by a pH-dependent Fe(II)-tartrate complex and the initial formation of H_2_O_2_; the following second phase is the more common “Fenton chemistry”, in which tartaric acid is oxidized by “Fenton reagents”, Fe(II) and H_2_O_2_.

Given that very little H_2_O_2_ (micromoles) is needed to initiate (and run) the second phase, in which a large quantity of substrate is oxidized, the second phase is autocatalytic, in which H_2_O_2_ is regenerated to keep the reaction going. Often in Fenton chemistry, it is hydrogen peroxide that is the main oxidant that determines the amount of substrate oxidized; here, very little H_2_O_2_ is needed, and the reaction itself regenerates H_2_O_2_, which makes the reaction autocatalytic, when oxygen is available.

Tartaric acid is unique as it promotes both oxygen activation to produce the initial H_2_O_2_ to “ignite” the reaction of oxidation and self-oxidation by generating more H_2_O_2_. The stoichiometry of autocatalysis is interesting—it is almost 1:1 in H_2_O_2_ consumed and regenerated, in a chain-like fashion, but not exactly so. About 1/10th of generated H_2_O_2_ is accumulated in the solution during the propagation stage. This indicates a nontrivial mechanism in which several reaction paths run in parallel and quantitatively give an apparent stoichiometry close to approximately one H_2_O_2_ consumed and one H_2_O_2_ regenerated.

The speed of propagation remains essentially constant over the course of the reaction, which means that it is zero-order in O_2_ and Fe(II), and in H_2_O_2_ as well; the concentrations of all these components change significantly, but the rate of propagation does not. It is obviously a chain reaction, a feature which is not unknown in Fenton chemistry;^31^ however, here, the rate of the reaction remains constant, which indicates a special stationary condition at the propagation stage. Earlier, we speculated that some intermediate or catalytic complex, which produces oxidation, is involved and serves as a bottleneck of the reaction.^27,28^ (Similar autoxidation patterns but with slower rates and less complete reaction have been observed with Fe(II) and malic or citric acid.^27^ One interpretation of these findings is that they result in a less efficient generation of hydrogen peroxide in the initiation stage.)

pH affects both the initiation and the propagation stages, but in the opposite manner: it speeds up the initiation and slows down the propagation. The initiation speedup can be understood on the basis of the need to form the Fe(II)-tartrate complex to reduce the redox potential of Fe(II) in the solution. The aqua-complexes will follow the trend, as more OH- ligands bound to Fe will be formed with increasing pH. However, the redox potential of aqua-Fe at very low pH is close to 0.7 V, which is too high to react directly with O_2_ and produce superoxide, O_2_·, as the redox potential of the latter is −0.16 V. Thus, given the redox potentials, it is more likely that the formation of the tartaric-iron complex is crucial for the initiation.

Moreover, given the relatively small amount of ionized tartaric acid at pH 2.5 (p*K*_a1_ is 2.8)^32^ and the shift of redox potential of Fe ions (around 0.3 to 0.4 V),^29,33^ the formation of free superoxide (protonated at our pH’s, p*K*_a_ 4.8)^34^ at the initiation stage is unlikely as the difference in redox potentials is still too high. Therefore, we assume formation of bi-nuclear complexes such as Fe(III)-OOH-Fe(II) to produce H_2_O_2_, completely by-passing the formation of free per-hydroxyl, ·OOH. This is supported by our experiments with SOD, which produced very little effect; in contrast, catalase produced a significant inhibition.^29^

### The Complete Reaction Description

The proposed mechanism of the observed kinetics is based on a radical propagation reaction initiated by either the high valence ferryl ion^35,36^ or the hydroxyl radical,^32^ as described elsewhere.^27,28^ The complete oxidation scheme (see the Supporting Information) can be reduced to the following reactions (1)–(7) shown in Table 1:

**Table 1:** Reduced Oxidation Scheme for the Reaction of Fe(II), Oxygen, and Tartaric Acid^a^

*k* values	units	reaction
1.4 × 10^–2^	M^–2^ s^–1^	(1) Fe(II) + O_2_ + RH_2_ → Fe(III) + RH• + H_2_O_2_
3.5 × 10^2^	M^–1^ s^–1^	(2) Fe(II) + H_2_O_2_ → Fe(*IV*)O^++^ + H_2_O
4.9 × 10^0^	M^–2^ s^–1^	(3) Fe(*IV*)O^++^ + 2 RH_2_ → Fe(II) + 2 RH•
2.5 × 10^7^	M^–2^ s^–1^	(4) RH• + O_2_ + Fe(II) → Fe(III) + H_2_O_2_ + R
1.4 × 10^1^	M^–1^ s^–1^	(5) RH• + Fe(III) → Fe(II) + R
9.3 × 10^–6^	M^–2^ s^–1^	(6) Fe(III) + RH_2_ + H_2_O_2_ → Fe(*IV*)O^++^ + RH• + H_2_O
9.6 × 10^1^	M^–1^ s^–1^	(7) RH• + RH• → RR

a RH_2_ = tartaric acid, RH• = tartaric radical, R = dihydroxymaleic acid (DHMA), RR = dimer.

**Figure 1:**
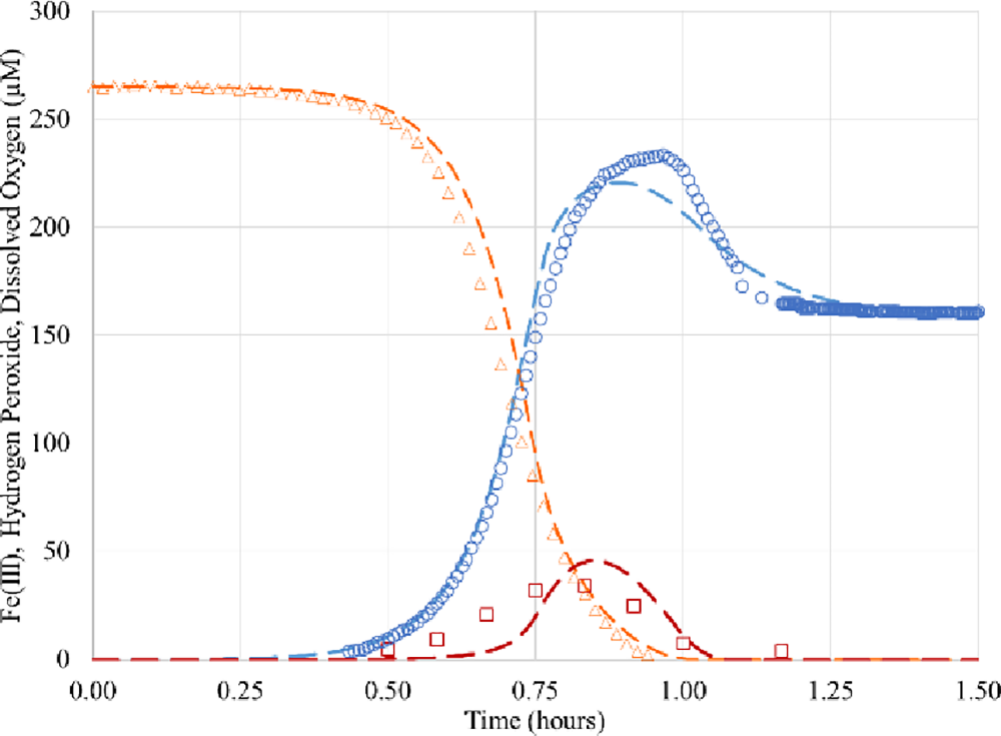
Experimental kinetic data and theoretical fits, using a scheme in Table 1. Dissolved oxygen (orange triangle)^29^ and predicted (orange dashed lines), Fe(III) (blue circle)^29^ and predicted (blue dashed lines), and hydrogen peroxide (red square)^29^ and predicted (red dashed lines).

This reaction scheme, when fitted the experimental data using the Kintecus software,^30^ yields the rate constants of individual reactions. In Table 1, data are shown for pH 2.5. Figure 1 shows the fitting for pH 2.5. Both, qualitatively and quantitatively, the fit is rather accurate. However, the complete set of equations in Table 1 by itself does not reveal the main driving forces of the observed kinetics. One obstacle is the multitude of reactions and their complicated nature. Our goal in the following is to simplify the description and elucidate the main driving factors of the reaction.

### The Reduced Reaction Description

It turns out that the equations of the complete scheme can be simplified. First, we find that reaction (6) can be neglected (due to the small rate constant and involvement in the termination stage). Second, reactions (2) and (3) can be approximately described as a combined reaction (a sum of (2) and (3)). The resulting simplified reaction scheme is shown in Table 2.

**Table 2:** Fully Simplified Scheme for the Reaction of Fe(II), Oxygen, and Tartaric Acid^a^

*k* values	units	reaction
6.1 × 10^–2^	M^–2^ s^–1^	(1) Fe(II) + O_2_ + RH_2_ → Fe(III) + RH• + H_2_O_2_
3.6 × 10^0^	M^–2^ s^–1^	(2) H_2_O_2_ + 2 RH_2_ → 2 RH• + 2 H_2_O
4.8 × 10^6^	M^–2^ s^–1^	(3) RH• + O_2_ + Fe(II) → Fe(III) + H_2_O_2_ + R
1.7 × 10^1^	M^–1^ s^–1^	(4) RH• + Fe(III) → Fe(II) + R
1.5 × 10^2^	M^–1^ s^–1^	(5) RH• + RH• → RR

a RH_2_ = tartaric acid, RH• = tartaric radical, R = dihydroxymaleic acid (DHMA), RR = dimer.

**Figure 2:**
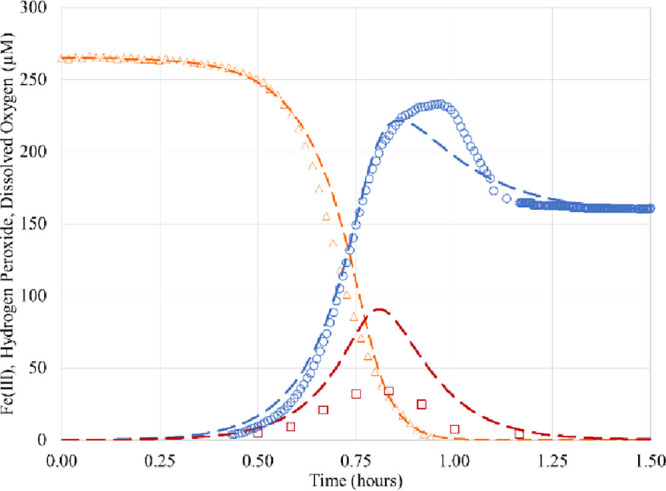
Experimental kinetic data and theoretical fits, using a further refined scheme in Table 2. Dissolved oxygen (orange triangle)^29^ and predicted (orange dashed lines), Fe(III) (blue circle)^29^ and predicted (blue dashed line), and hydrogen peroxide (red square)^29^ and predicted (red dashed lines).

Using this simplified kinetic scheme, the fitting to experimental data is still of reasonable quality and results in rate constants shown in Table 2 (at pH 2.5, same as Table 1). Figure 2 shows the resulting model fit. The fitted hydrogen peroxide maximum in this approximate scheme is about twice the value observed; however, this is not important for the analysis that follows.

The following analysis of the simplified scheme in Table 2 will reveal the nontrivial nature of the driving forces of the observed kinetics. But the key feature can already be seen in the proposed simplified scheme. Given the observed 1:1 stoichiometry of Fe(II)/O_2_, combining reaction (2) and two reactions (3) from Table 2, the autocatalytic propagation reaction condenses to the to the following equation:
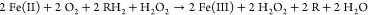
1.1

This equation describes the exponential amplification of hydrogen peroxide. Each cycle of the reaction generates two hydrogen peroxides for every one entering the cycle. This chain reaction would continue indefinitely, if it were not for the terminating/dissipating reactions (4) and (5) in Table 2. The rates of reactions (4) and (5) relative to that of the condensed eq 1.1 define the condition of the exponential growth. The overall kinetic character is defined by the competition of multiplication and termination reactions.

To quantify the above multiplication process, we focus on the key variables of the reaction and re-write the kinetic scheme in terms of pseudo-first-order reactions for these variables. This is the key idea of the analysis. Following this, we first introduce the pseudo-first-order rate constants *k̅_i_* for the equations in Table 2:

(1) Fe(II) + O_2_ + RH_2_ → Fe(III) + H_2_O_2_ + RH·
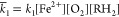


(2) H_2_O_2_ + 2RH_2_ → 2RH· + 2H_2_O
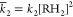


(3) RH· + O2 + Fe(II) → Fe(III) + H_2_O_2_ + R
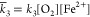


(4) RH· + Fe(III) → Fe(II) + R
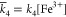


(5) RH· + RH· → dimer
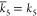


Here, R is dihydroxymaleic acid (DHMA). It should be noticed that reactions (2) and (3) result in the multiplication of H_2_O_2_ mentioned earlier.

In order to explore the condition of exponential growth, we consider a simplified reduced description of the system, keeping track of the most important variables: hydrogen peroxide (*h*), tartaric acid radicals (*r*), oxygen, and Fe(III). Using pseudo-first-order rate constants, the kinetics of hydrogen peroxide and tartaric acid radicals can be written as follows:
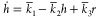
1.2
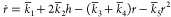


As mentioned earlier, here, two tartaric acid radicals RH· are generated for one H_2_O_2_ consumed in the proposed scheme.

Two additional equations of interest are for oxygen and for Fe(III):
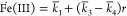
1.3
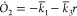


The pseudo-first-order reaction rate constants *k̅_i_* change in time together with the concentrations of the key reaction components, as defined by eqs (1)–(5) in the above scheme; however, at a given stage of the reaction, its kinetic character can be determined by performing the exponential (Lyapunov) stability analysis^37^ of linearized system described next.

### Lyapunov Stability of the Reaction System. Exponential Growth of Radicals

The linear part of the coupled equations that determines the character of kinetic behavior of the system has the form
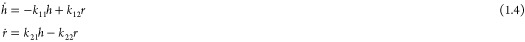


Here, *k*_11_ is a combined rate of conversion of hydrogen peroxide to hydroxyl radical and to ferryl complexes and also decomposition of hydrogen peroxide by Fe(II); *k*_12_ is the rate of regeneration of hydrogen peroxide by the reaction of tartaric acid radicals with oxygen; *k*_21_ is the rate of generation of tartaric acid radicals (it may not be exactly the same as *k*_11_); *k*_22_ is the rate of tartaric acid radical removal due to oxidation by Fe(III) (and generation of DHMA).

The stability of the kinetic system is defined by the above linearized equations and its kinetic matrix K_ij_ = *k*_11_, *k*_12_, *k*_21_, *k*_22_. The kinetics is bi-exponential; the two rates are given by the eigenvalues of the kinetic matrix found from the following equation:
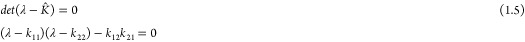
The populations are changing as combination of two exponentials:
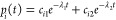
1.6where *c_i_* are some constants.

When the product *k*_12_*k*_21_ = 0, the two eigenvalues are λ_1_ = *k*_11_ and λ_2_ = *k*_22_. The two rates describe bi-exponential relaxation of hydrogen peroxide and tartaric acid radicals to their equilibrium values. However, when *k*_12_*k*_21_ > 0, one eigenvalue may become negative. In this case, the negative eigenvalue gives rise to an exponential growth (and the propagation phase of the reaction).

For our reduced pseudo-first-order system, eq 1.2, the negative eigenvalue is given by
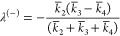
1.7

If *k̅*_3_ < *k̅*_4_, there is no negative eigenvalue. But if *k*_3_ is larger *k*_4_, the eigenvalue is negative, indicating exponential growth—or inflation of radicals in the system. We can now look at our kinetic data. Recall that pseudo-first-order rates *k̅_i_* are themselves functions of varying concentrations and depend on time; thus, the above condition is expected to vary with time.

### Stability Analysis at pH 2.5

**Figure 3:**
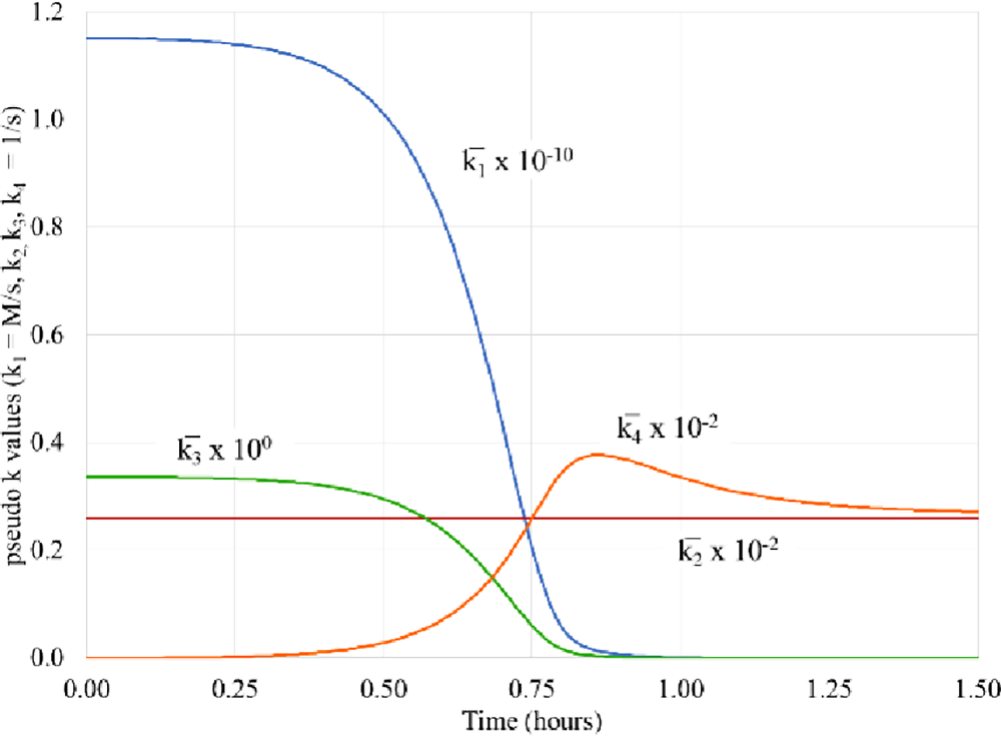
Pseudo-first-order rates *k̅_i_* at pH 2.5.

At pH 2.5, the pseudo-first-order rates *k̅_i_* are shown in Figure 3.

It is seen that here that the rate constant *k̅*_3_ is largest; moreover, *k̅*_3_ ≫ *k̅*_4_ and hence the negative eigenvalue is approximately given by
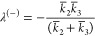
1.8that is, it is determined by the smallest of *k̅*_2_ and *k̅*_3_. Since *k̅*_2_ is about 2.5 × 10^–3^ s^–1^, it is the smallest (*k̅*_3_ ≈ 10^–1^ s^–1^), thus, it gives the negative eigenvalue. This corresponds to a timescale of exponential growth of about 10^3^ s, which is exactly the initiation time of the reaction.

When the exponential growth of hydrogen peroxide and tartaric acid radicals begins, the dissipation/termination processes get activated and (quasi) stationary concentrations are quickly established; this will continue until oxygen and Fe(II) are diminished. At low pH, the condition of exponential growth is satisfied up to very low concentrations of oxygen; eventually, of course, it breaks down, as *k̅*_3_, the rate of regeneration of hydrogen peroxide for which oxygen is needed, diminishes to zero, but *k̅*_4_, the rate of removal of radicals, increases with increasing Fe(III).

The initial lag, before the fully developed propagation stage, is due to a very small rate of production and concentration of hydrogen peroxide initially. Accumulation of hydrogen peroxide in the system and subsequent exponential growth of radicals, with their stabilization by the termination processes, *k̅*_5_, give rise to a stationary propagation phase of oxidation. The latter is observed as almost linear dependence of oxygen consumption in the fully developed stationary propagation stage.

### Stability Analysis at pH 4.5

**Figure 4:**
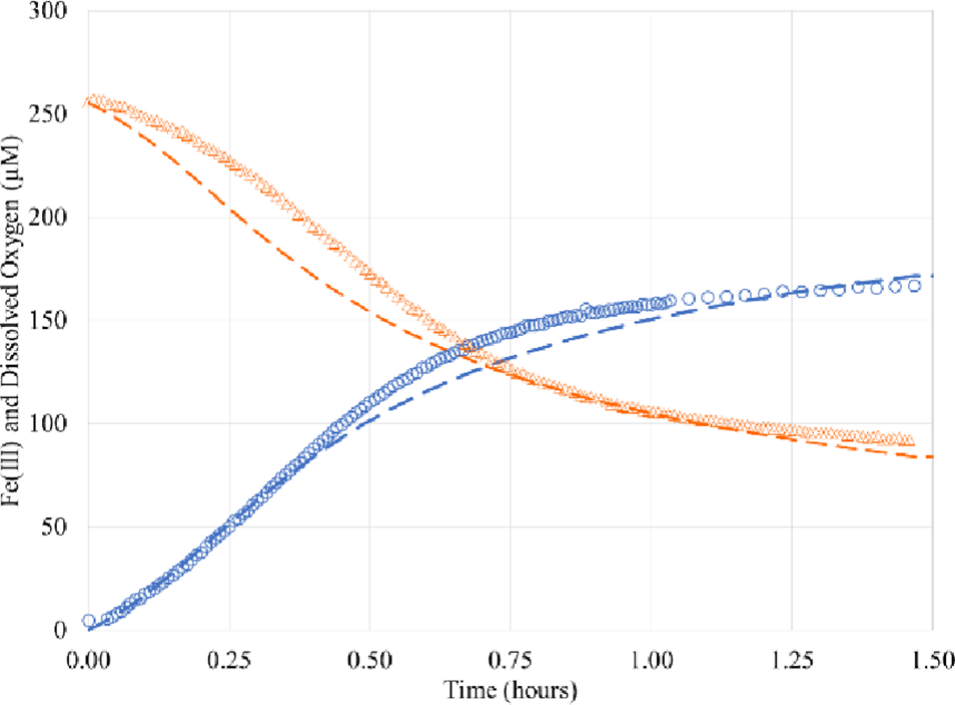
Experimental kinetics and theoretical fits at pH 4.5, using a further refined scheme in Table 2. Dissolved oxygen (orange triangle)^29^ and predicted (orange dashed lines), and Fe(III) (blue circle)^29^ and predicted (blue dashed lines).

At higher pH 4.5, the kinetics are completely different, as shown in Figure 4. There is almost no stationary propagation phase. This qualitative change of kinetics can be explained in terms of the properties of the chain reaction of radicals.

At high pH, the multiplication and exponential growth of radicals, the reason for a stationary propagation phase at pH 2.5, is obviously no longer present. This can occur in two cases: (1) when *k̅*_3_ < *k̅*_4_ and, hence, there is no negative eigenvalue, according to eq 1.7 and hence no exponential growth. Another possibility is that (2) *k̅*_3_ > *k̅*_4_ and the negative eigenvalue is formally still present, but it is so small that the exponential phase has no time to develop, before other factors would lead to termination of the reaction. We expect that at higher pH one of these two mechanisms will suppress the exponential growth phase. Which is the case for our reaction?

**Figure 5:**
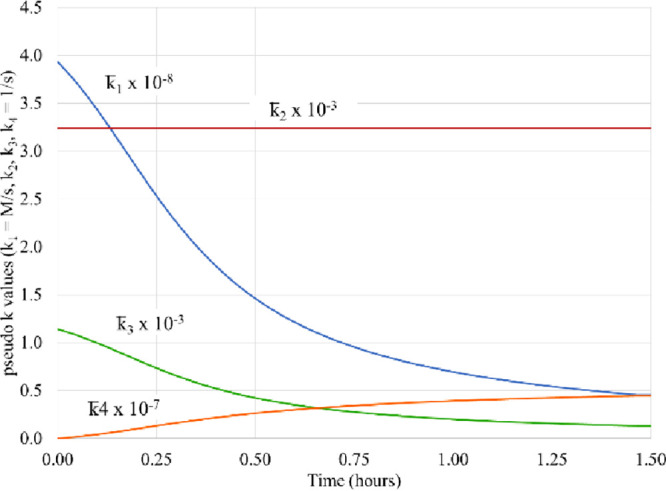
Pseudo-first-order rates *k̅_i_* at pH 4.5.

The pseudo-first-order reaction rates for pH 4.5 are shown in Figure 5. Here, the rate *k̅*_4_ is still very small so that *k̅*_3_ > *k̅*_4_, and thus, the negative eigenvalue is still present. However, the rate *k̅*_3_ itself is now very small, about hundred times smaller than that at pH 2.5, and smaller than *k̅*_2_. As we know from eq 1.7, when *k̅*_3_ ≫ *k̅*_4_, the negative eigenvalue is determined by the smallest of *k̅*_2_ and *k̅*_3_. Now at high pH, *k̅*_3_ is much smaller than *k̅*_2_, in contrast to low pH, and thus, according to eq 1.7, the negative eigenvalue is defined by *k*_3_, λ^( – )^ ≃ *k̅*_3_. During the reaction, the pseudo-first-order rate *k*_3_ is decreasing, and on the reaction time scale, it is so small that it appears to be irrelevant. In this case, the exponential phase here would not have a chance to develop, and thus, no inflationary growth of radicals occurs at high pH.

Indeed, a more rigorous analysis, see Appendix, indicates that the exponential growth phase occurs only when the so-called exponential growth factor θ_exp_, the inflation parameter, is large. This parameter is a measure of the role of different rates in the kinetics of the system and is given by
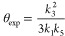
1.9

This factor is a quantitative measure of the inflationary growth of radicals in the oxidation kinetics,
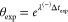
1.10during the exponential growth time, Δ*t*_exp_.

If θ_exp_ ≃ 1, there is no exponential growth of radicals. It turns out that this is indeed the case at pH 4.5 (θ_exp_^4.5^ ≃ 1). But if θ_exp_ ≫ 1, which is the case of pH 2.5 (θ_exp_^2.5^ ≈ 10^3^), there is an exponential growth of radicals. In this case, it quickly brings the rate of oxidation to its maximum value, and after stabilization by the radical dimerization reaction (*k*_5_), it results in an almost constant rate of oxygen consumption—the propagation phase, shown in Figure 1 at pH 2.5.

In summary, at high pH, the negative eigenvalue that describes the rate of exponential growth becomes too small compared to other relevant rates (*k*_1_ and *k*_5_), so that the stationary propagation phase of oxidation does not develop. There is a qualitative change of the character of the reaction with increasing pH, which resembles critical behavior.

### Analogy with Phase Transitions

The critical behavior of the kinetics in our system, as a function of pH, is qualitatively similar to that of some statistical systems. Namely, if the two principal concentrations *h* and *r* are considered to be dynamic variables that move on their free energy surface, along the gradients of the surface, as
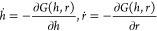
1.11then the free energy function is given by a quadratic form
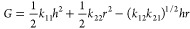
1.12

This scheme results in a symmetric kinetic matrix; in order to return to the original general kinetic scheme eq 1.4, one needs to rescale the variables as follows: , 

The eigenvalues of the kinetic matrix define the curvature along the principal axis of the above free energy surface. When both eigenvalues are positive, the free energy surface is a stable paraboloid, with equilibrium of both variables at zero. Dynamically, whatever the initial condition, the two variables will tend to zero as time advances. However, when one eigenvalue is negative, the free energy surface at the origin is a saddle point and in one direction becomes unstable. Dynamically, the two variables will deviate from their initial values with time, increasing in their absolute values (which corresponds to exponential growth of radical concentrations).

Qualitatively, the change of behavior as a function of pH can be described as a typical second-order phase transition with the free energy of the system of the form (*a* > 0, *B* > 0):
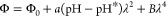
1.13

Here, λ represents the exponential growth rate (the negative eigenvalue of the kinetic matrix), which is defined by the minimum of the above free energy. At pH > pH*, the minimum corresponds to zero value of λ (i.e., no exponential growth); but when pH < pH*, the minimum of free energy is at a nonzero value of λ (i.e., exponential growth). Such critical behavior of λ as a function of pH is typical for second-order phase transitions.

In our case, the transition to a new (kinetic) phase at pH < pH* describes the appearance of self-sustained chain reaction in the system. The “critical” value of pH in our system is around 3.0–3.5,^27,28^ so that at pH 2.5, there is a well pronounced propagation phase, while at pH 4.5, it is practically absent.

However, as shown in the Appendix, more rigorously the presence or absence of the exponential growth of radicals is described by the inflation parameter η = ln θ_exp_. Therefore, in the above phenomenological free energy instead of rate λ, one should better use a dimensionless parameter η = ln θ_exp_. The “transition” then is a change from η = 0 at high pH > pH* to η > 0 at low pH < pH*, which indicates the exponential inflation phase of radicals at low pH, see Appendix for additional details.

## Conclusions

In this and the previous recent reports, we have described new details of tartaric acid autoxidation that point to a pH-dependent formation of catalytic complexes of metal and tartrate ions in which oxidation takes place. These data extend our understanding of the molecular mechanism of this classic reaction that has played such a prominent role in chemistry in the past. Although several chelating substrates can be autoxidized in the presence of Fe(II),^38^ the chain-like oxidation that regenerates H_2_O_2_ and runs with constant speed appears to be a unique feature of tartaric acid autoxidation. Most of the earlier proposed models of Fenton oxidation appear to have overlooked this self-propagating chain reaction and the role of Fe(II) complexes in its initiation.

Here, we have shown that the propagation phase of autoxidation is a chain reaction that regenerates hydrogen peroxide with positive feedback; this is a typical mechanism for self-sustained chain reactions. The condition for such chain behavior is shown to be similar to critical behavior, when the kinetic matrix of pseudo-first-order reaction becomes negatively defined, which signals exponential growth of key reactive intermediates in the system. The exponential growth exists at low pH 2.5 and practically absent at high pH 4.5, with a critical pH value around 3.5.

The kinetic stability analysis of a simplified reaction mechanism presented in this work provides insights into the earlier experimental outcomes of the autoxidation of tartaric acid, across the pH region 2.5 to 4.5.

One implication of the present work is that some of the oxidation reaction systems that have been studied previously may have been initiated by the mechanism described here. Another is that other autocatalytic reactions in this mildly acidic pH range, especially those involving hydrogen peroxide, may have analogous stability behavior and critical pH for propagation.

Fenton oxidation plays a central role in oxidative processes in living cells. The low pH conditions such as pH 4.5 can occur in lysosomes. It would be interesting to look further and see if it is possible to find conditions for chain oxidation at higher pH than at pH 2.5 observed here. Controlled oxidation of this nature would find many interesting applications.

## A Appendix

### A.1 Exponential Growth Factor, θ_exp_

In the limiting cases *k*_2_ ≫ *k*_3_ and *k*_2_ ≪ *k*_3_, a simple analytical treatment of the reduced kinetics equations eq 1.2 is possible. Shown are some results of such an analysis.

To simplify notation, we will write *no bars* for the rates, *k_i_*, and consider the case of *k*_3_ ≪ *k*_4_, which corresponds to both high and low pH. In this case, the equations are

A.1

To avoid unnecessary complications, we will further assume that the rates *k*_1_–*k*_5_ do not depend on time, although a more general treatment is also possible.

Consider the case of *k*_2_ ≫ *k*_3_, which corresponds to pH 4.5. The equations for hydrogen peroxide, *h*, and radicals, *r*, can be viewed as describing a dynamic system (considering *h* and *r* as “velocities)” with friction forces −*k*_2_*h* and −*k*_3_*r*. When *k*_2_ is large, the large friction −*k*_2_*h* results in a stationary state for *h*, *ḣ* = 0, which gives the relation between hydrogen peroxide, *h*, and tartaric radicals, *r*

A.2

which in turn gives one closed equation for *r*, which is easy to analyze

A.3

Initially, when radical concentration *r* is small, the growth of radicals is linear in time, with the rate of 3*k*_1_, *r* ≈ 3*k*_1_*t*. When concentration reaches first critical value, *r*_1_^*^ = 3*k*_1_/*k*_3_, the exponential growth begins, with the rate *k*_3_. We notice that according to a general result eq 1.7, when *k*_2_ ≫ *k*_3_, the exponential growth rate is λ^( – )^ ≃ *k*_3_, which is reproduced here. The exponential growth phase can be approximated as

A.4

This growth will continue until a second critical value of radical concentration is reached, ; this is a maximum radical concentration that is determined by the (quadratic) termination reaction *k*_5_. The exponential phase, inflation, therefore is

A.5

The growth factor θ_exp_ is now defined as

A.6

which is given by

A.7

This factor reflects the “inflation” of radicals produced by the initiation rate *k*_1_, during the exponential growth phase. A similar analysis of the opposite case results in essentially the same estimate.

At pH 4.5, the substitution of typical values of *k*_1_, *k*_3_, and *k*_5_ gives, θ_exp_^4.5^ ≃ 1 and pH 2.5, θ_exp_^2.5^ ≃ 10^3^.

Of course, the rates should be remembered to vary in time but using the *typical* values of rates during the reaction gives the correct quantitative picture, as the comparison of direct numerical integration with the above estimates shows.

The above estimates show *qualitative* difference in kinetics at pH 2.5 and pH 4.5. One is characterized by the exponential growth or inflation of radicals after initiation, and the other shows no exponential inflation.

As the equation for oxygen evolution shows, when the exponential growth is present (at pH 2.5), the rate of oxygen combustion is changing during the initiation period from *k*_1_ to a maximum *k*_3_*r*_2_^*^ = *k*_3_*r*_1_^*^θ_exp_ and does not essentially change after that (radical dimerization - termination processes limits the exponential growth). This results in the oxidation phase (propagation) that occurs with maximum rate, *k*_3_*r*_2_^*^. Thus, the consumption of oxygen after initiation of the reaction occurs approximately linearly with time, with maximum rate, *k*_3_*r*_2_^*^. Numerical integration of kinetic equations confirms this qualitative picture.

The qualitative change with pH can be formally described as a kinetic “phase transition” if we write a phenomenological Gibbs energy of our system in terms of exponential growth parameter η = ln θ_exp_ as follows:

A.8

with (*a*, *B* > 0). When pH is above “critical” value pH*, the minimum of Gibbs energy (as a function of η) corresponds to η = 0. However, when pH < pH*, the minimum of Gibbs energy is at a nonzero value of η, and

A.9

The nonzero value of η is related to the rate of oxygen combustion during the propagation phase of Fenton chain oxidation.

### A.2 Exact Solution

For constant rates, the system allows for an exact analytical solution, which further clarifies the kinetics of the system. For example, for the above case *k*_3_ ≪ *k*_2_, the equation for radical evolution has the following (almost) exact solution:

A.10

Here, for simplicity, we assumed  (i.e., to be large, or somewhat larger than unity), otherwise this ratio should be replaced with unity.

It is seen that for small *t* ≤ *k*_3_, the radicals grow linearly, as expected, as *r* ≃ *k*_1_*t*, until the first critical value *r*_1_^*^ = *k*_1_/*k*_3_. After that, if the maximum value *r*_2_^*^ = *k*_3_/*k*_5_ is not reached, the growth continues exponentially, as *r*_1_^*^exp(*k*_3_*t*), until the maximum value *r*_2_^*^ = *k*_3_/*k*_5_. The exponential growth factor is θ_exp_ ≃ *k*_3_^2^/*k*_1_*k*_5_. However, if *r*_2_^*^ ≤ *r*_1_^*^, the exponential growth does not occur, as the maximum value is already reached during the linear stage. Thus, as we saw previously, the signature of the presence of exponential inflation of radicals is the condition θ_exp_ = *k*_3_^2^/*k*_1_*k*_5_ ≥ 1.

The oxygen evolution occurs as follows: Ȯ_2_ = – (*k*_1_ + *k*_3_*r*). Initially, the radical concentration is small, and oxygen is consumed linearly with time with the rate *k*_1_. If exponential growth of radicals is present, the rate quickly increases to its maximum value of *k*_max_^O_2_^ = *k*_3_*r*_2_^*^ = *k*_3_^2^/*k*_5_, and further oxygen combustion occurs again linearly with time but with much increased rate. In the opposite case of no radical inflation, the rate of oxygen consumption increases quadratically up to a value (*k*_1_ + *k*_3_*r*_1_^*^), which is only about twice as large as *k*_1_, and constant after that.

The above picture should be modified, as we need to recall that all rates are themselves functions of concentrations. In particular, *k*_3_ ∝ [O_2_][Fe^2+^] ∝ [O_2_]^2^, i.e., has very strong (quadratic) dependence. If we recall the exponential growth factor θ_exp_ = *k*_3_^2^/*k*_1_*k*_5_, it is seen that it depends on oxygen concentration as [O_2_]^4^ and, thus, drops very quickly with oxygen consumption. This dependence has two effects: first, it limits the growth rate in the exponential phase as oxygen is consumed, and second, the stationary states predicted by the constant rate theory should be modified in a self-consistent manner, now predicting a decrease of corresponding stationary values as rates decrease with oxygen and Fe(II) consumption. If this is all taken into account, the above analytic theory accurately reproduces all the observed experimental kinetic data, as the comparison with the exact numerical integration of kinetic equations shows.

A similar solution can be found in the opposite case *k*_3_ ≫ *k*_2_. In this case, *k*_2_ plays the role of the negative eigenvalue and that of the rate of exponential expansion. Here, the first initiation interval is *t* ≤ 1/*k*_2_, and by the end of initiation, the radical concentration *r*_1_^*^ ≃ 3*k*_1_/*k*_3_; the further evolution is defined by whether or not the limiting value of radicals, here *r*^max^ = *r*_2_^*^ ≃ *k*_3_/2*k*_5_, is reached. If the maximum of radicals is not reached during initiation, there will be further exponential growth of radicals up to the maximum value, and exponential growth factor, θ_exp_ = *k*_3_^2^/6*k*_1_*k*_5_. If, on the other hand, the maximum is already reached during initiation, there will be no exponential expansion, which is formally indicated by the growth factor, θ_exp_ ≃ 1.

It is seen that the difference with the previous *k*_3_ ≪ *k*_2_ case is the rate of exponential growth, which is the smallest of *k*_2_ and *k*_3_, in agreement with our eq 1.7. Finally, it should be noticed that the negative eigenvalue λ^( – )^ of eq 1.7 also defines the initiation time, Δ*t*_in_ = 1/λ^( – )^.

## Supporting Information Available

Fitting parameters for complete reaction scheme (PDF)

## Dedication

This paper is dedicated to Rudy Marcus on the occasion of his 100th birthday.
