# Supplementary material for: Chain Reaction of Fenton Autoxidation of Tartaric Acid: Critical Behavior at Low pH
Source: J Phys Chem B. 2023 May 10;127(19):4300–8. doi: 10.1021/acs.jpcb.3c02172 (PMC10201527; doi:10.1021/acs.jpcb.3c02172)
Supplement: Supplementary file 1 — jp3c02172_si_001.pdf [file jp3c02172_si_001.pdf]

Supplementary Information for:

Chain Reaction of Fenton Autoxidation of Tartaric Acid: Critical Behavior at Low pH

Robert E. Coleman,[†] Roger B. Boulton[†], and Alexei A. Stuchebrukhov[‡]

[†]Department of Viticulture and Enology and [‡]Department of Chemistry, University of California, Davis, CA 95616, United States

Corresponding Author: aastuchebrukhov@ucdavis.edu

1. The detailed chemical scheme of Fenton autoxidation of Tartaric acid (RH_2)

The reaction was modeled using the following scheme:

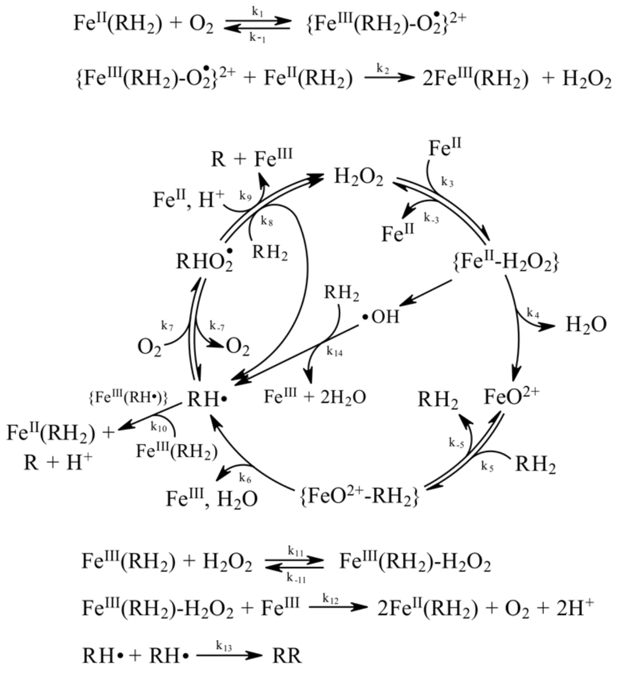

Scheme S1. Mechanism used for the modeling of the oxidation of tartaric acid (RH_2) revised from Ref. [29] to include the decomposition of hydrogen peroxide by Fe(III)(RH_2) in reactions (11) and (12).

Reactions (1) and (2) are initiation reactions producing initial H_2O_2. Reactions (11) and (12) are the reverse of (1) and (2). An alternative reaction for (1) and (2) is oxidation of one Fe(II) and one RH2: Fe(II)(RH_2)+ O_2 + (RH_2) → Fe(III)(RH_2) + H_2O_2 + RH· , which gives similar fitting results as shown in Figures below. Of critical importance is reaction (8).

2. Fitting parameters and fitting results for complete reaction scheme.

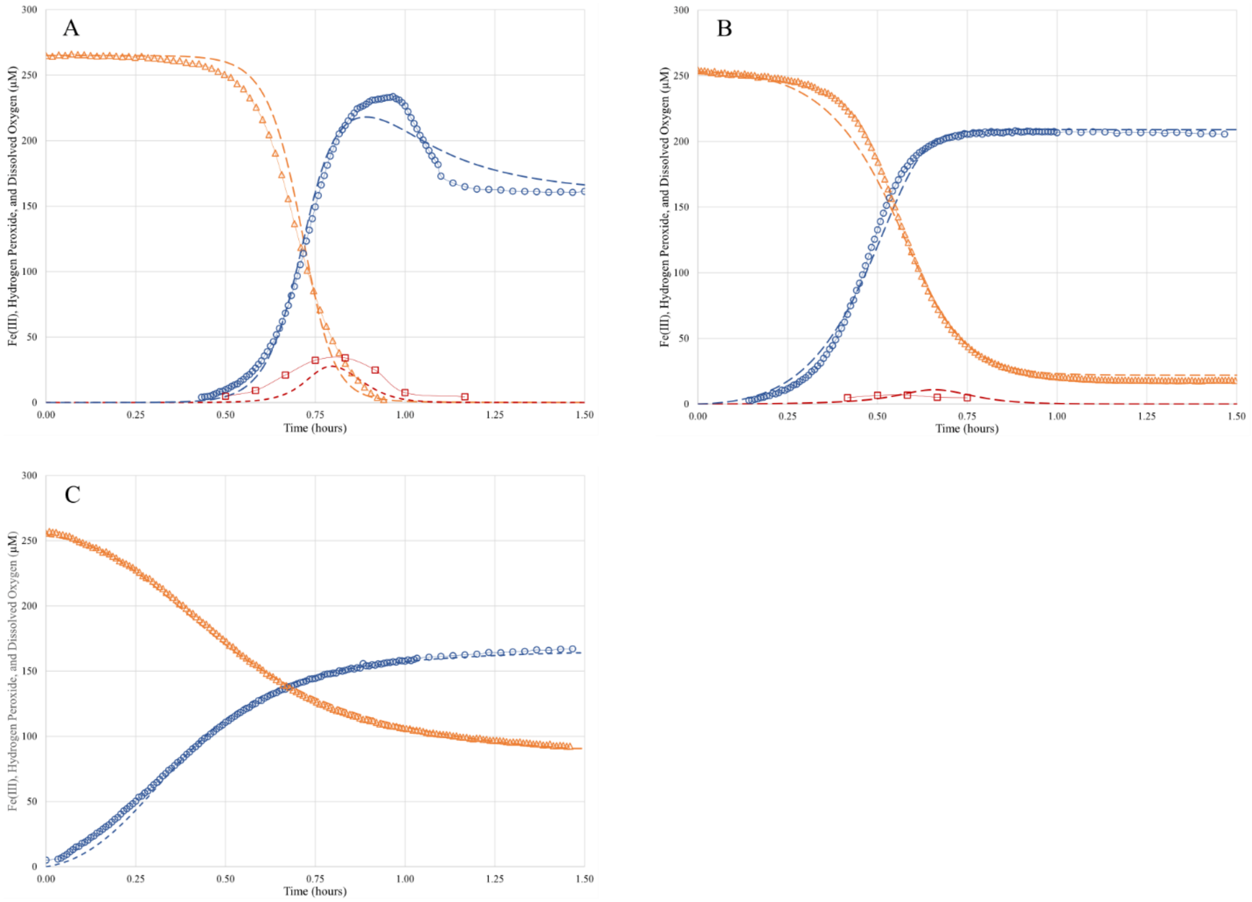

Figure S1. Fitting results. Dissolved oxygen (△)[29] and predicted (···), Fe(III) (○)[29] and predicted (···), and hydrogen peroxide (□)[29] and predicted (···) time traces modeled with dissolved oxygen, Fe(III), and hydrogen peroxide time traces at 265 μM initial Fe(II) in air-saturated 26.7 mM tartaric acid at (A) pH 2.5, (B) pH 3.5 and (C) pH 4.5.

Table S1. Estimated kinetic constants for 265 µM initial Fe(II) in air-saturated 26.7 mM tartaric acid at pH 2.5 for Scheme 1.

k_1/k_{-1}	5.3E+01	M^{-1}
k_2	1.3E+01	$M^{-1}s^{-1}$
k_3/k_{-3}	1.5E+04	M^{-1}
k_4	1.3E-02	s^{-1}
k_5/k_{-5}	4.0E+02	M^{-1}
k_6	4.9E+05	s^{-1}
k_7/ k_{-7}	7.8E+01	M^{-1}
k_8	1.4E+02	$M^{-1}s^{-1}$
k_9	4.8E+03	$M^{-1}s^{-1}$
k_{10}	6.6E+00	$M^{-1}s^{-1}$
k_{11}/ k_{-11}	1.0E+00	M^{-1}
k_{12}	1.1E+02	$M^{-1}s^{-1}$
k_{13}	3.7E+00	$M^{-1}s^{-1}$
